# Transcriptomic characterization of the functional and morphological development of the rumen wall in weaned lambs fed a diet containing yeast co-cultures of *Saccharomyces cerevisiae* and *Kluyveromyces marxianus*

**DOI:** 10.3389/fvets.2025.1510689

**Published:** 2025-01-22

**Authors:** Zixuan Xu, Lan Yang, Hui Chen, Pengxiang Bai, Xiao Li, Dacheng Liu

**Affiliations:** College of Veterinary Medicine, Inner Mongolia Agricultural University, Hohhot, China

**Keywords:** weaned lamb, transcriptome, co-cultures, oxidative phosphorylation, rumen

## Abstract

**Introduction:**

In lambs, the function of the rumen is incompletely developed at weaning, and the inclusion of yeast cultures in the diet can profoundly influence the morphological and functional development of the rumen.

**Methods:**

In this study, the effects of *Saccharomyces cerevisiae* and *Kluyveromyces marxianus* (NM) yeast co-cultures on ruminal histomorphology were assessed, and corresponding transcriptomic changes within the rumen epithelium were identified. In total, 24 lambs were grouped into four groups of six lambs including a control (C) group fed a basal diet, and N, M, and NM groups in which lambs were fed the basal diet, respectively, supplemented with *Saccharomyces cerevisiae* yeast cultures (30 g/d per head), *Kluyveromyces marxianus* yeast cultures (30 g/d per head), and co-cultures of both yeasts (30 g/d per head), the experiment lasted for 42 d.

**Results:**

In morphological analyses, lambs from the NM group presented with significant increases in papilla length, papilla width, and epithelial thickness in the rumen relative to lambs in the C group (*p* < 0.05). Transcriptomic analyses revealed 202 genes that were differentially expressed between samples from the C and NM groups, with the largest proportion of these genes being associated with the oxidative phosphorylation pathway. In a weighted gene coexpression network analysis, a positive correlation was observed between the MEgreen and MEpurple modules and rumen morphology. Of these modules, the MEgreen module was found to be more closely linked to fatty acid metabolism and oxidative phosphorylation, whereas the MEpurple module was linked to oxidative phosphorylation and fatty acid degradation. Ultimately, these results suggest that dietary supplementation with NM has driven the degradation of fatty acids, the induction of oxidative phosphorylation, the acceleration of lipid metabolism, the production of ATP to sustain ruminal growth, and the maintenance of intracellular NADH/NAD+ homeostasis on weaned lambs and is superior to single yeast fermentation.

**Discussion:**

These results thus offer a theoretical foundation for further studies examining the mechanisms through which NM cultures can influence ruminal development in lambs.

## 1 Introduction

The weaning process is a critical stage in lamb development, and the rumen plays a central role in the coordination of the uptake and use of nutrients in weaned lambs (1), with an appropriately developed rumen being vital to metabolic activity, immune function, and overall health (2). The rumen epithelium is composed of lobular papillae that facilitate absorption while also presenting an epithelial barrier that can provide protection against the entry of toxins or microbes from the rumen (3). Rumen epithelial morphology is central to the ways in which ruminants respond to changes in dietary composition (4). The development of this epithelial layer, in turn, is influenced by a wide variety of factors that include dietary physico-mechanical stimulation (5) as well as chemical stimulation mediated by the products of feed fermentation (6). Yeast cultures have been reported to influence the composition of the rumen microflora in lambs, stimulating their overall growth and enhancing immunological activity (7, 8). As they can be stored easily and or of high levels of economic value, these cultures are often used for the preparation of feed (9). These cultures can alter the morphology of the rumen epithelium in weaned lambs as a result of shifts in the expression of epithelial genes (10). The impacts of different strains of yeast culture on the rumen epithelial barrier, however, differs markedly (11). Research shows that higher levels of overall rumen epithelial thickness have been observed for goats fed *S. cerevisiae* yeast cultures, with changes in the expression of the cyclin D1 gene having been linked to increases in teat growth (12, 13). Adding *Bacillus subtilis* and *S. cerevisiae* co-cultures has been shown to increase ruminal gene expression related to proliferative activity while reducing expression levels linked to the process of VFA uptake (14). The addition of different yeast cultures may have specific effects on the molecular processes that shape rumen functional and morphological characteristics in ruminants after weaning.

RNA sequencing (RNA-seq) is a high-throughput transcriptomic analytical technique that can offer abundant quantitative and qualitative insights into activities in eukaryotic or prokaryotic cells and associated conditions (15, 16). Such approaches have been used extensively when analyzing rumen development (17). Zhuang et al. (18) for instance, demonstrated that *β*-hydroxybutyric acid supplementation resulted in increases in rumen papillae length and width, and they further identified hub genes enriched in the nicotinamide adenine dinucleotide (NADH) dehydrogenase (ubiquinone) activity Gene Ontology (GO) pathway. Baldwin et al. (19) also performed transcriptomic studies of rumen samples from Holstein cows prior to and at 14, 42, 56, and 70 days after weaning, revealing a shift in the reliance of the rumen epithelium from glucose toward VFA as a source of energy. Sun et al. (20) demonstrated that introducing fermentation concentrates led to the enhancement of fatty acid and amino acid metabolism within the rumen in pre-weaned lambs. Introducing hay together with ferment concentrate preparations resulted in the activation of rumen epithelial growth and development-related gene pathways in lambs (21). Additionally, the altered thickness of the rumen epithelium has been found to be associated with a range of cellular functions including proliferative activity (22) and the differentiation and proliferation of the epithelium (23). Transcriptionally, rumen epithelial morphology has been found to be correlated with particular gene-targeted functions including cellular development (24), the proliferation of the epithelium (25), papilla size and surface area, and tight junctions (26). Many recent studies have shown that co-culture can provide benefits to animals, such as supplementation with *Kluyveromyces marxianus* and *Pichia kudriavzevii* co-cultures that improve feed intake and milk quality in cows, Co-culture with *Lactobacillus rhamnosus* and *Saccharomyces cerevisiae* increases the content of short-chain fatty acids in mammal guts (27, 28). *S. cerevisiae* and *K. marxianus* cultures are both rich sources of metabolites (29), but there has been little research exploring how these two yeast species can modulate the development of the rumen epithelium.

As such, in this study, differentially expressed genes were identified in the rumen epithelium of lambs fed NM yeast cultures through a transcriptomics approach, ultimately enabling the identification of pathways associated with particular rumen epithelial features. Overall, these analyses provide insight into how dietary NM supplementation can influence the morphology of the rumen epithelium and the associated molecular mechanisms.

## 2 Materials and methods

### 2.1 Yeast culture preparation

The natural fermented mare’s milk was filtered and diluted in the laboratory, and then separated and purified in the yeast extract peptone glucose medium (YPD). *S. cerevisiae* and *K. marxianus* strains with good fermentation characteristics were identified, pre-grown at 30°C on YPD for 24 h, and inoculated into the fermentation medium, and pilot production efforts were conducted at Kehong Feed Co., Ltd. (Inner Mongolia, China). The basal diet for lambs in this study was composed of 12% bran, 12% spraying corn bran, 10% corn, 10% rice bran, 10% cottonseed meal, 8% wheat shorts, 28% corn germ meal, and 10% soybean meal. *S. cerevisiae* (3 × 10^8^ CFU/g), *K. marxianus* (3 × 10^8^ CFU/g), or a 1: 1 mixture of the two (3 × 10^8^ CFU/g) were used to inoculate the diet at 8% per 1,000 kg wet mixed matrix through the addition of sterile water with constant stirring for a final system moisture content level of 40%. Aerobic fermentation at 30°C was then performed for 72 h. The nutrient compositions of the yeast cultures are presented in Supplementary Table S1 (30).

### 2.2 Experimental design and diet

The experiments in this study were performed at Fuchuan Farm in Bayannaoer, Inner Mongolia, China. In total, 24 healthy (23.5 ± 2.85 kg) 2-month-old weaning (Duber × thin-tail sheep) lambs were randomly assigned to four groups (*n* = 6/group) while maintaining comparable average body weight levels in each of these groups. The animals in the control (C) group were fed a basal diet, while those in the N, M, and NM groups were, respectively, fed diets supplemented with fermentation cultures of *S. cerevisiae*, *K. marxianus,* or a combination of the two (30 g/head/day). These lambs were fed at 08: 00 and 19: 00 daily in equal amounts. The study was performed over the course of 42 days, with the first 7 being the pre-test period. Animals had free food and water access during this time. The composition and nutrient content in the basal diet are presented in Table 1.

**Table 1:** Dietary composition and basal diet nutrient content (%, DM basis).

Ingredients	C	N	M	NM
Peanutvine	9.30	9.30	9.30	9.30
Corn stalk	10.00	10.00	10.00	10.00
Sunflower seed skin	4.00	4.00	4.00	4.00
Alfalfa meal	10.00	10.00	10.00	10.00
Corn grain	29.00	29.00	29.00	29.00
Soybean meal	6.00	6.00	6.00	6.00
Germ meal	8.00	8.00	8.00	8.00
Cottonmeal	6.00	6.00	6.00	6.00
DDGS Distillers Dried Grains with Solubles	6.00	6.00	6.00	6.00
Sunflower Cakes	7.50	7.50	7.50	7.50
NaCl	0.50	0.50	0.50	0.50
Limestone	0.5	0.5	0.5	0.5
CaHPO4	0.50	0.50	0.50	0.50
4%Premix^1^	2.7	2.7	2.7	2.7
Total	100.00	100.00	100.00	100.00
Nutrient levels				
ME, MJ/kg	10.19	10.21	10.18	10.22
Dry matter %	89.77	88.25	88.13	87.75
Crude protein %	15.62	16.23	15.98	16.85
Neutral detergent fiber %	33.35	34.03	33.45	33.38
Acid detergent fiber %	21.40	20.99	21.38	21.59

1 The premix contained the following (per kg): Fe 60 mg, Cu 12 mg, Zn 60 mg, Mn45 mg, nicotinic acid 60 mg, I 0.6 mg, Se, 0.2 mg, vitamin A, 3500 IU, vitamin D, 1200 IU, vitamin E, 20 IU, Ca 2 g, P 1 g, Co 20 mg, NaCl 5 g.

C = basal diet; N = *Saccharomyces cerevisiae* yeast cultures; M = *Kluyveromyces marxianus* yeast cultures; NM = *Saccharomyces cerevisiae* and *Kluyveromyces marxianus* co-cultures yeast cultures.

### 2.3 Analyses of the ruminal epithelium

After the 42-day feeding period, animals were harvested before the morning feeding at 7: 00. The rumen was then harvested and separated from the omasum as in prior reports (31). Previous methods were then used to conduct a detailed characterization of the histomorphology of the rumen papillae (32). Briefly, the ventral rumen sac was chosen as it has been found to exhibit the highest mucous capillary blood flow per unit weight of any portion of the rumen (33). Sterile surgical scissors were used to collect 800 mg of rumen tissue, which was then washed with cold PBS (pH 7.2) 20 times. Then, ~2 cm^2^ samples of rumen epithelial tissue including rumen atria, ventral rumen sac, and ventral caecum were collected, fixed with 4% paraformaldehyde overnight, dehydrated, paraffin-embedded, cut into 8 μm sections, and stained with a conventional hematoxylin and eosin (H&E) approach. Six slices were produced for each lamb rumen and repeated three times. Papillae of similar size and shape from paraffin sections were selected for histomorphological analyses, analyzing the morphological features of these papillae from four sections with the optimal papillary orientation being in the median sagittal plane. Analyses were conducted with Image-Pro Plus 6.0 (Media Cybernetics Inc., Bethesda, MD, USA). Rumen papilla width and length were measured as reported previously (32). Rumen papillae were also separated from the basal epithelial layer, snap-frozen in liquid nitrogen, and stored at −80°C in the lab for subsequent transcriptomic analyses.

### 2.4 Transcriptomic sequencing

TRIzol (Tiangen, Beijing, China) was used for the isolation of RNA, followed by the use of a NanoDrop spectrophotometer (Agilent, Cary 60 UV–Vis, CA, USA) and 1% agarose gel electrophoresis assay to measure RNA quantity and integrity. Then, 3 μg of total RNA was selected as input for sequencing library preparation. Initially, magnetic beads were used to purify the mRNA, followed by its fragmentation at high temperatures using divalent cations in fragmentation buffer from Illumina. SuperScript II and random oligonucleotides were then employed for first-strand DNA preparation, with DNA polymerase I and RNase h being used for second-strand cDNA preparation. Nucleic acid cleavage enzymes and polymerase activity were then used to convert any overhangs into blunt ends, followed by the removal of these enzymes. Next, 3′ adenylation was performed, followed by ligation of the Illumina PE adapter oligonucleotides in preparation for hybridization. Fragments of cDNA that were 400–500 bp long were selected through the use of the AMPure XP system (Beckman Coulter, CA, USA) for purification. Selective enrichment of DNA fragments for junction molecules attached at both ends was achieved through a 15-cycle PCR reaction with an Illumina PCR primer cocktail. After using the AMPure XP system to purify the resultant products, they were subjected to high-sensitivity DNA analysis-based quantification with a Bioanalyzer 2100 system (Agilent). A NovaSeq 6000 platform (Illumina) was used for sequencing, which was performed by Shanghai personalbio biotechnology Co. Ltd. (Shanghai, China). An in-house Perl script was used for the removal of low-quality reads. Then, HISAT2 (34) was used to align the remaining clean reads with the *Ovis aries* reference genome (Oar v3.1). StringTie (version 1.3.4d) was used for assembly (35) and the fragments per kilobase per million mapped fragments (FPKM) values were calculated to evaluate the expression of gene transcripts. The Personalbio Cloud Platform^1^ was used to compile volcano plots, and Gene Ontology (GO) functional classification and Kyoto Encyclopedia of Genes and Genomes (KEGG) pathway enrichment analyses were performed. In addition, KOBAS (version 2.0) was utilized to evaluate the significant enrichment of differentially expressed genes (DEGs) in KEGG pathways (36).

### 2.5 qRT-PCR verified the expression of genes associated with transcriptomic data

The results of transcriptomic analyses were validated by selecting 9 DEGs to analyze via qPCR. TRIzol (Tiangen) was used for the extraction of RNA from samples of ruminal epithelium. Primer designs were generated in Primer Premier 5 (Table 2), with GADPH as a reference control. Each qPCR reaction (20 μL) included 2 × ChamQ Universal SYBR qPCR Master Mix (10 μL), cDNA template, and forward and reverse primers (each 0.4 μL at 10 μM), and thermocycler settings were: 95°C for 30 s, and cycles of 95°C for 5 s, 60°C for 20 s, and GAPDH served as a normalization control. Amplicon specificity was confirmed through a melt curve analysis, and relative expression was assessed via the 2^−ΔΔCt^ method (37).

**Table 2:** Primers sequences used for real-time PCR.

Gene bank ID	Gene	Primers (sense/antisense 5′-3′)	Amplicon size
XM_027956625.3	*ACADS*	Forward: ATTGTGCTGTGAACTACGC	150 bp
		Reverse: GCCAACTTGAACTGGATGAC	
XM_004020520.5	*ATP5F1A*	Forward: GGGACCCTATGTACTCGGA	172 bp
		Reverse: GATAGGGCAGTGAAGGTCTC	
XM_027980034.3	*CCNB1*	Forward: GATTGGAGAGGTTGATGTCG	116 bp
		Reverse: TGCACCATGTCATAGTCCA	
XM_027957175.2	*COX5A*	Forward: CAGATGAGGAGTTTGATGCT	278 bp
		Reverse: TGTGTTTATCCCTTTACGCA	
XM_042234178.1	*MAPK1*	Forward: CTATTTGCTTTCTCTTCCACAC	176 bp
		Reverse: CAATAAGTCCAGAGCTTTGGA	
XM_015104158.3	*MAPK3*	Forward: AGACTCCAAAGCCCTTGAC	148 bp
		Reverse: GGTCATAGTACTGCTCCAGG	
XM_042235740.2	*MYD88*	Forward: GTTATTAAGAAGGACTGGAGAAAGG	186 bp
		Reverse: AATCCTCCTCTGAAAGCCTC	
XM_060407205.1	*NDUFA1*	Forward: TTGGGTATCACTGGAGTCTG	173 bp
		Reverse: TTCTCCAAACCCTTTGACAC	
XM_027972913.2	*NDUFB9*	Forward: CGATTGCTACAAGGTCCCA	166 bp
		Reverse: TCTCTTGGCAAAGTAATCAGGA	
XM_060411593.1	*GAPDH*	Forward: TGGCATCGTGGAGGGACTTA	60 bp
		Reverse: CATCATACTTGGCAGGTTTCTCC	

### 2.6 Statistical analyses

Differences in parameters pertaining to the ruminal epithelium were compared between groups via one-way ANOVAs in SPSS 25.0 (SPSS Inc., IL, USA), with *p* < 0.05 defining significance. The weighted gene co-expression network analysis (WGCNA) algorithm in the R package was used to access the modules of genes related to the characterization of the rumen epithelium development and different groups. The qRT-PCR data were analyzed using one-way ANOVA for expression levels in each group and compared using Tukey’s honestly significant difference test (Tukey’s HSD). *p* < 0.05 was considered to be statistically significant. For the analysis of gene expression data, RNA-Seq data were initially subjected to log2 transformation to approximate a normal distribution better and stabilize the variance. The False Discovery Rate (FDR) was calculated using the Benjamini-Hochberg method to control the rate of false positives in the context of multiple comparisons. Mann–Whitney tests were then employed to compare log2-transformed FPKM values between groups. The fold-change cutoff for identifying differentially expressed genes (DEGs) was set at >1.2, which, in the context of log2-transformed data, this choice was made to balance sensitivity and specificity in detecting biologically meaningful changes while controlling for multiple comparisons. DEGs identified when comparing the C vs. N, C vs. M, C vs. NM, N vs. NM and M vs. NM groups were detected based on this fold-change >1.2 in the log2-transformed data and an FDR < 0.05.

## 3 Results

### 3.1 Morphology of the rumen epithelium

**Figure 1:**
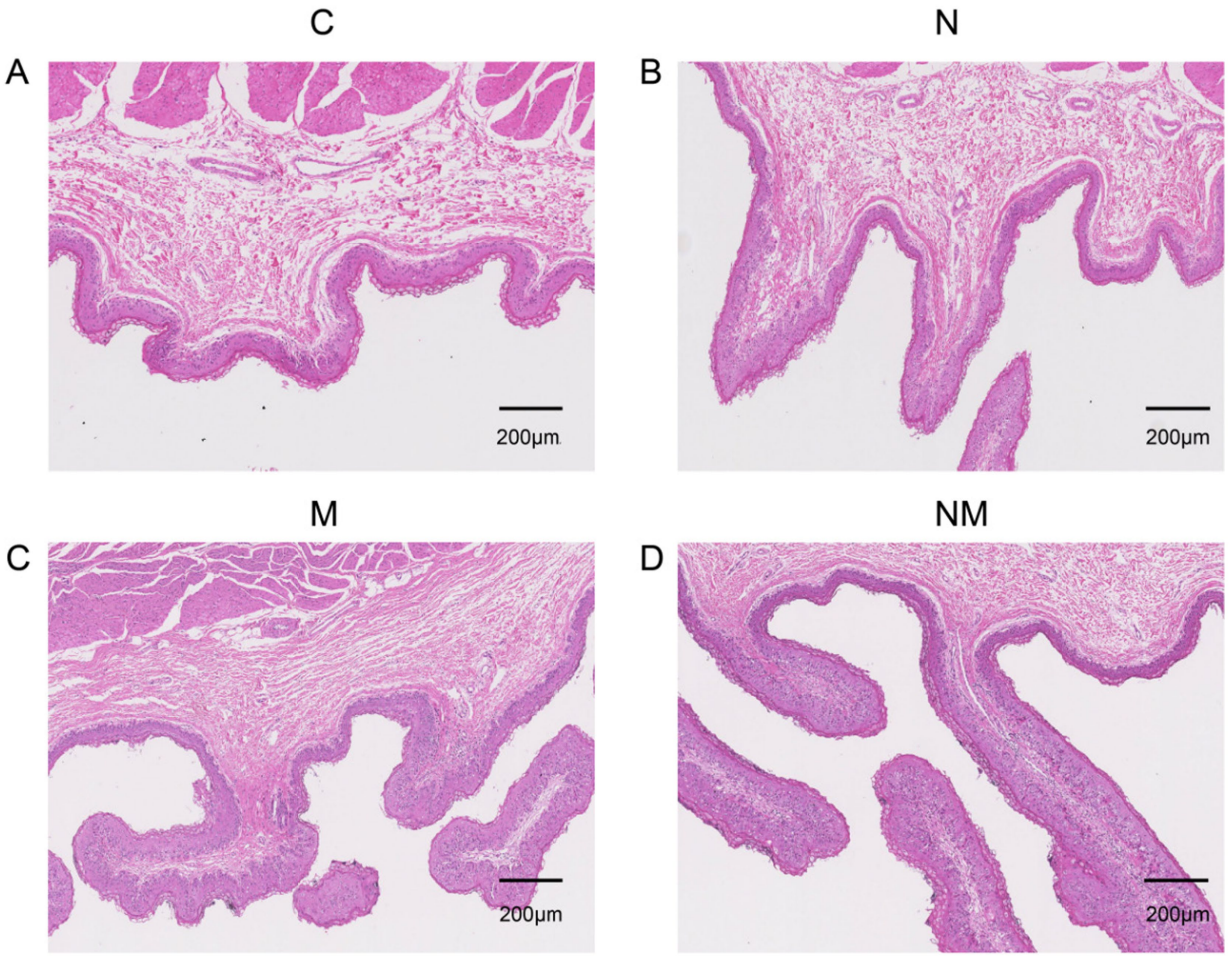
The impact of dietary N, M, and NM on ruminal epithelial characteristics in lambs. Representative rumen epithelium images are shown for comparisons for groups **(A)** Basal diet **(B)** *Saccharomyces cerevisiae* yeast cultures **(C)** *Kluyveromyces marxianus* yeast cultures **(D)** NM = *Saccharomyces cerevisiae* and *Kluyveromyces marxianus* co-cultures yeast cultures.

Rapid, healthy ruminal development is essential for weaned lamb growth and development. Accordingly, the development of the rumen in weaned lambs from the NM group was characterized, revealing several changes in morphological features in the epithelium attributable to yeast supplementation (Figure 1 and Table 3). The rumen papilla lengths were significantly greater in the NM group compared to the control (C) group (*p* = 0.01). N, M, and NM supplementation also led to significant increases in the width of these papillae (*p* = 0.002). Significant increases in the thickness of the rumen epithelium were evident in the N, M, and NM groups relative to the C group (*p* < 0.001), and significantly greater thickness was evident in the NM group relative to either of the individual yeast supplementation groups. Lamina propria thickness was unchanged across groups (*p* = 0.05). These data thus highlighted the ability of NM supplementation to promote rumen papillary development.

**Table 3:** The impact of dietary N, M, and NM supplementation on ruminal epithelial characteristics in lambs.

Epithelial morphology	Groups				SEM	*p*-value
	C	N	M	NM		
Papilla length, μm	932.74^b^	1252.45^ab^	1250.13^ab^	1619.31^a^	85.484	0.010
Papilla width, μm	308.68^c^	327.47^b^	330.35^ab^	335.47^a^	7.562	0.002
Lamina propria thickness, μm	353.92	356.74	349.28	403.01	8.431	0.051
Epithelial thickness, μm	50.19^c^	60.37^b^	62.86^b^	72.69^a^	2.613	<0.001

^a, b, c^ Values in a row with distinct superscripts differ significantly (*p* < 0.05). Data are means and SEM (standard error of the mean).

C = basal diet; N = *Saccharomyces cerevisiae* yeast cultures; M = *Kluyveromyces marxianus* yeast cultures; NM = *Saccharomyces cerevisiae* and *Kluyveromyces marxianus* co-cultures yeast cultures.

### 3.2 RNA-seq analysis and DEG identification

**Figure 2:**
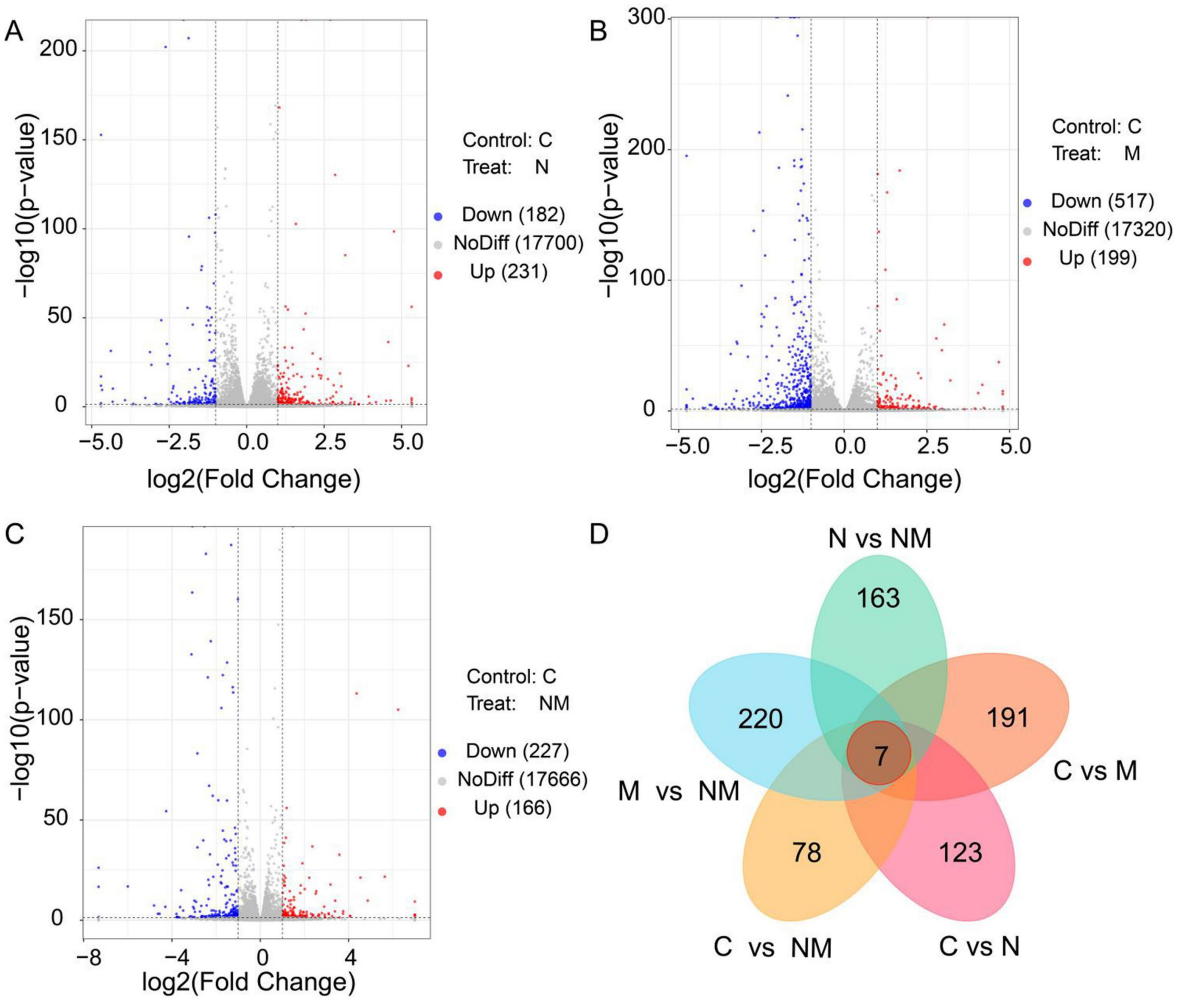
Differentially expressed gene identification in the ruminal epithelium for the three different group comparisons. **(A)** C vs. N, **(B)** C vs. M, and **(C)** C vs. NM. In the generated volcano plots, the *X*-and *Y*-axis, respectively, represent log2 fold change and log10 *p*-values, with blue, red, and gray dots indicating downregulated DEGs, upregulated DEGs, and non-DEGs, respectively. **(D)** A Venn diagram of the overlap in DEGs for the indicated comparisons. C = basal diet; N = *Saccharomyces cerevisiae* yeast cultures; M = *Kluyveromyces marxianus* yeast cultures; NM = *Saccharomyces cerevisiae* and *Kluyveromyces marxianus* co-cultures yeast cultures.

Next, efforts were made to explore the effects of NM supplementation on gene expression in the ruminal epithelium. These analyses yielded 79.00 GB of clean data, with an average of 6.58 GB per sample. The expression levels for 17.032 genes were measured across samples. Relative to the C group, the N group exhibited 413 DEGs (231 upregulated, 182 downregulated) (Figure 2A), the M group exhibited 716 DEGs (199 upregulated, 517 downregulated) in group M (Figure 2B), and the NM group exhibited 393 DEGs (166 upregulated, 227 downregulated) (Figure 2C). Relative to the NM group, the N group exhibited 490 DEGs (198 upregulated, 292 downregulated), and the M group exhibited 759 DEGs (538 upregulated, 221 downregulated) (Supplementary Figures S1A,B). Venn diagrams also revealed 7 DEGs shared between these comparisons, whereas there were 123, 191, 78, 163 and 220 unique DEGs, respectively, associated with the C vs. N, C vs. M, C vs. NM, N vs. NM, and M vs. NM comparisons (Figure 2D).

### 3.3 Functional enrichment analyses

To gain additional insight into the shifts in biological activity that may related to the DEGs from the NM group, GO and KEGG enrichment analyses were next performed for the DEGs identified for the C vs. N, C vs. M, C vs. NM, N vs. NM, and M vs. NM comparisons.

**Figure 3:**
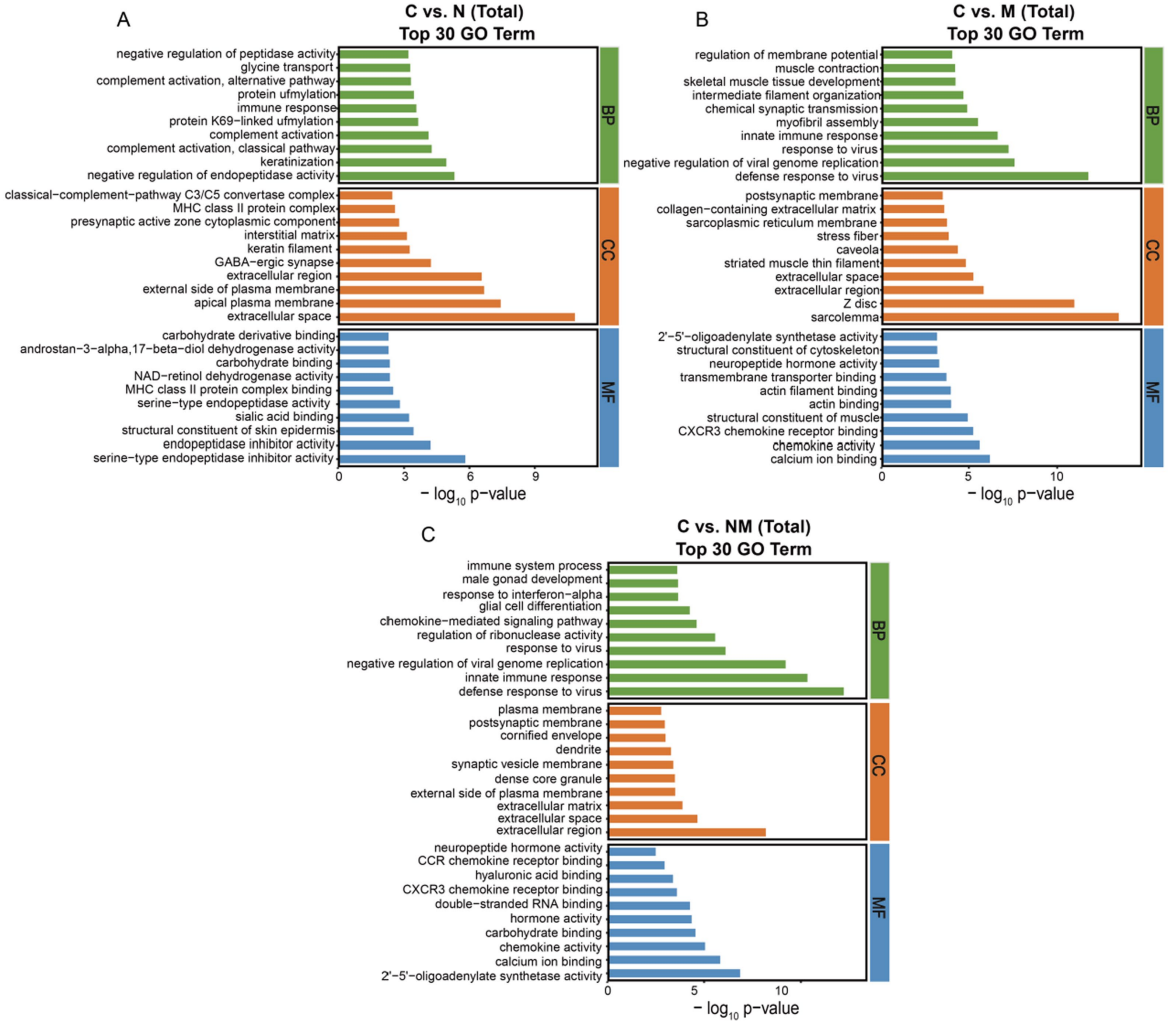
Analyses of DEG enrichment in specific GO terms. The top 30 biological process, cellular component, and molecular function terms are shown (*p* < 0.05; the DEGs number of GO terms was >2). **(A)** C vs. N. **(B)** C vs. M. **(C)** C vs. NM. C = basal diet; N = *Saccharomyces cerevisiae* yeast cultures; M = *Kluyveromyces marxianus* yeast cultures; NM = *Saccharomyces cerevisiae* and *Kluyveromyces marxianus* co-cultures yeast cultures.

For GO analyses (Figure 3), the C vs. N DEGs were associated with biological process (BP) terms including “negative regulation of endopeptidase activity” and “keratinization,” cellular component (CC) terms including “extracellular space” and “apical plasma membrane,” and molecular function (MF) terms including “serine-type endopeptidase inhibitor activity” and “endopeptidase inhibitor activity” (Figure 3A). For the C vs. M comparison, DEGs were enriched in BP terms including “defense response to virus” and “negative regulation of viral genome replication,” CC terms including “sarcolemma” and “Z disc,” and MF terms including “calcium ion binding” and “chemokine activity” (Figure 3B). For the C vs. NM comparison, the DEGs were enriched in BP terms including “defense response to virus” and “innate immune response,” CC terms including “extracellular region” and “extracellular space,” and MF terms including “2′-5′-oligoadenylate synthetase activity” and “calcium ion binding” (Figure 3C). For the N vs. NM comparison, the DEGs were enriched in BP terms including “negative regulation of viral genome replication” and “defense response to virus,” CC terms including “extracellular space,” and MF terms including “protein-arginine deiminase activity.” For the M vs. NM comparison, the DEGs were enriched in BP terms including “chemical synaptic transmission” and “myofibril assembly,” CC terms including “Z disc,” and MF terms including “transmembrane transporter binding”(Supplementary Figures S2A,B).

**Figure 4:**
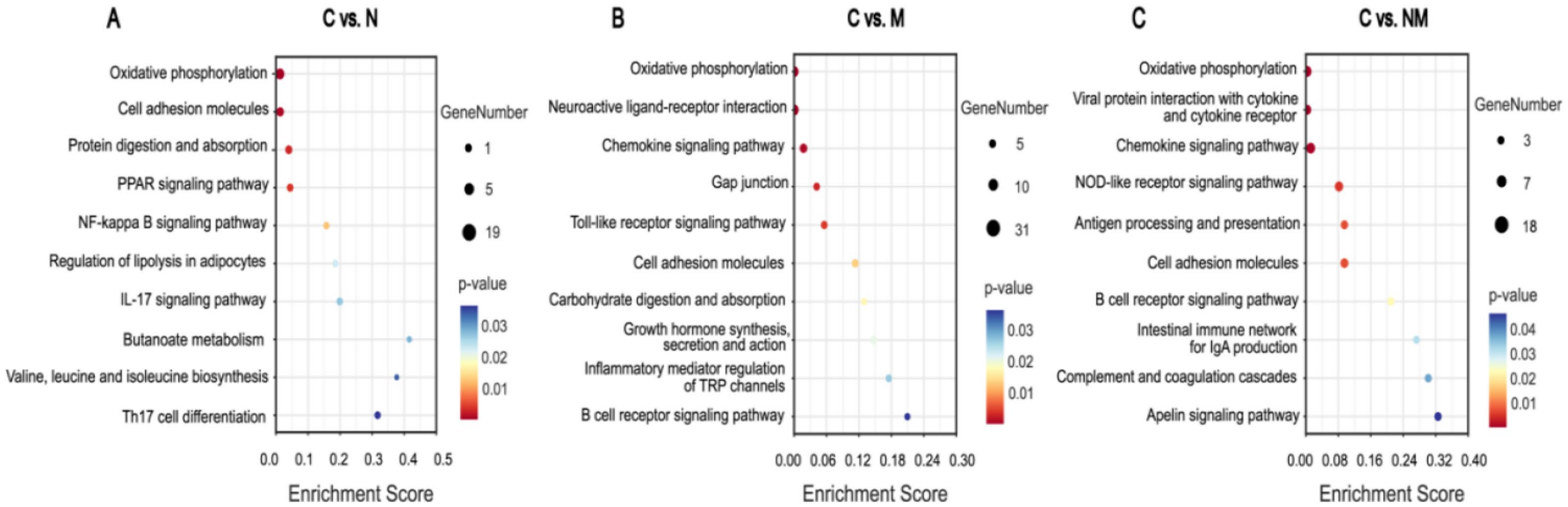
Analysis of DEG enrichment in KEGG pathways. Pathways and enrichment scores are, respectively, shown on the *Y*-and *X*-axes. Bubble sizes and colors correspond to significance levels and number of genes in each pathway, respectively (*p* < 0.05). **(A)** C vs. N. **(B)** C vs. M. **(C)** C vs. NM. C = basal diet; N = *Saccharomyces cerevisiae* yeast cultures; M = *Kluyveromyces marxianus* yeast cultures; NM = *Saccharomyces cerevisiae* and *Kluyveromyces marxianus* co-cultures yeast cultures.

For the C vs. N comparison, the oxidative phosphorylation, cell adhesion molecules, and protein digestion and absorption KEGG pathways exhibited significant enrichment (*p* < 0.05) (Figure 4A). For the C vs. M comparison these enriched pathways included the oxidative phosphorylation, neuroactive ligand-receptor interaction, and chemokine signaling pathways (*p* < 0.05) (Figure 4B). For the C vs. NM comparison they included the oxidative phosphorylation, viral protein interaction with cytokine and cytokine receptors, and chemokine signaling pathways (*p* < 0.05) (Figure 4C). For the N vs. NM comparison, the complement and coagulation cascades, protein digestion and absorption, and oxidative phosphorylation KEGG pathways exhibited significant enrichment (*p* < 0.05) (Supplementary Figure S3A). Lastly, for the M vs. NM comparison, the calcium signaling pathway, cell adhesion molecules and dopaminergic synapse KEGG pathways exhibited significant enrichment (*p* < 0.05) (Supplementary Figure S3B). Given its consistent enrichment, the oxidative phosphorylation pathway was selected for further analysis.

### 3.4 DEGs associated with the oxidative phosphorylation pathway

For the C vs. N comparison, 8 upregulated DEGs in the oxidative phosphorylation pathway were evident (*NDUFA1*, *NDUFS4*, *NDUFB9*, *COX15*, *COX5A*, *ATP5F1A*, *ATP5PB* and *ATP5F1C*) while 5 were downregulated (*NDUFV3*, *NDUFV2*, *UQCRH*, *UQCRFS1*, and *ATP5F1B*). Similarly, for the C vs. M comparison, there were 8 upregulated (*NDUFA1*, *NDUFV3*, *NDUFA10*, *NDUFA6*, *SDHA*, *COX5A*, *ATP5F1A*, and *ATP5F1B*) and 5 downregulated (*NDUFS4*, *NDUFV2*, *UQCRQ*, *COX15*, and *ATP5F1C*) DEGs in this pathway. Lastly, for the C vs. NM comparison, 16 upregulated DEGs (*NDUFA1*, *NDUFB9*, *NDUFA10*, *NDUFA12*, *NDUFV1*, *NDUFV2*, *SDHA*, *UQCRH*, *UQCRQ*, *UQCRFS1*, *COX15*, *COX5A*, *ATP5F1A*, *ATP5F1B*, *ATP12A*, *ATP4A*) that were associated with this pathway were identified (Table 4).

**Table 4:** DEGs associated with oxidative phosphorylation.

Gene ID	FPMK C vs. N	FPMK C vs. M	FPMK C vs. NM	Description
*NDUFA1*	Up	Up	Up	NADH:Ubiquinone Oxidoreductase Subunit A1
*NDUFS4*	Up	Down	Nodiff	NADH:Ubiquinone Oxidoreductase Subunit S4
*NDUFB9*	Up	Nodiff	Up	NADH:Ubiquinone Oxidoreductase Subunit B9
*NDUFV3*	Down	Up	Nodiff	NADH:Ubiquinone Oxidoreductase Subunit V3
*NDUFA10*	Nodiff	Up	Up	NADH:Ubiquinone Oxidoreductase Subunit A10
*NDUFA6*	Nodiff	Up	Nodiff	NADH:Ubiquinone Oxidoreductase Subunit A6
*NDUFA12*	Nodiff	Nodiff	Up	NADH:Ubiquinone Oxidoreductase Subunit A12
*NDUFV1*	Nodiff	Nodiff	Up	NADH:Ubiquinone Oxidoreductase Subunit V1
*NDUFV2*	Down	Down	Up	NADH:Ubiquinone Oxidoreductase Subunit V2
*SDHA*	Nodiff	Up	Up	Succinate Dehydrogenase Complex Flavoprotein Subunit A
*UQCRH*	Down	Nodiff	Up	Ubiquinol-Cytochrome C Reductase Hinge Protein
*UQCRQ*	Nodiff	Down	Up	Ubiquinol-Cytochrome C Reductase Complex III Subunit VII
*UQCRFS1*	Down	Nodiff	Up	Ubiquinol-Cytochrome C Reductase, Rieske Iron–Sulfur Polypeptide 1
*COX15*	Up	Down	Up	Cytochrome C Oxidase Assembly Homolog COX15
*COX5A*	Up	Up	Up	Cytochrome C Oxidase Subunit 5A
*ATP5F1A*	Up	Up	Up	ATP Synthase F1 Subunit Alpha
*ATP5PB*	Up	Nodiff	Nodiff	ATP Synthase Peripheral Stalk-Membrane Subunit B
*ATP5F1C*	Up	Down	Nodiff	ATP Synthase F1 Subunit Gamma
*ATP5F1B*	Down	Up	Up	ATP Synthase F1 Subunit Beta
*ATP12A*	Nodiff	Nodiff	Up	ATPase H+/K+ Transporting Non-Gastric Alpha2 Subunit
ATP4A	Nodiff	Nodiff	Up	ATPase H+/K+ Transporting Subunit Alpha

C = basal diet; N = *Saccharomyces cerevisiae* yeast cultures; M = *Kluyveromyces marxianus* yeast cultures; NM = *Saccharomyces cerevisiae* and *Kluyveromyces marxianus* co-cultures yeast cultures.

### 3.5 Weighted gene co-expression network analyses of the correlative relationship between host transcriptional features and indicators of ruminal epithelial development

**Figure 5:**
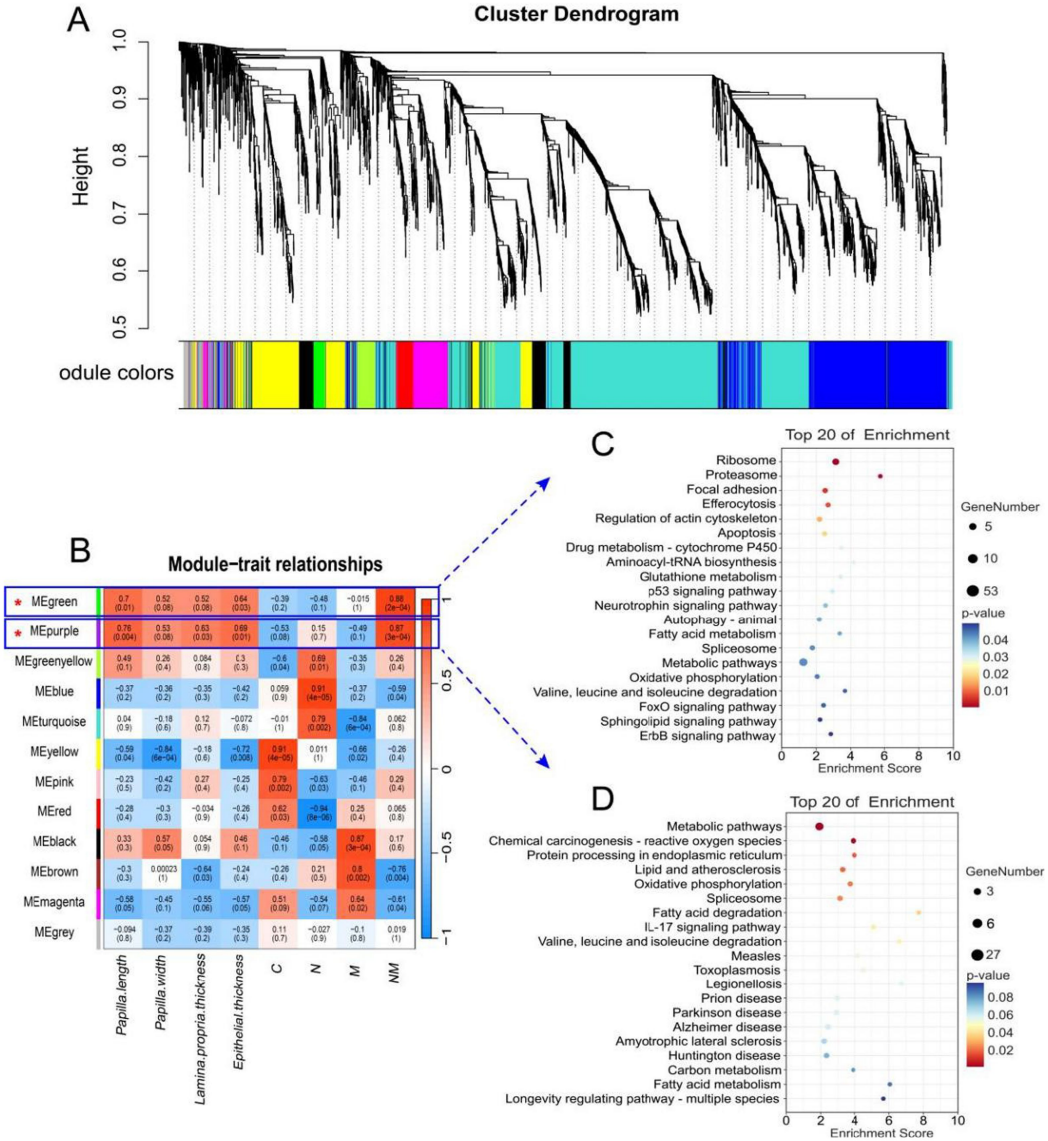
WGCNA identification of rumen tissue gene modules correlated with the rumen wall morphological and development indexes, and KEGG analysis of significant module. **(A)** The dendrogram derived from the gene co-expression network of samples in the four groups (C, N, M, and NM). **(B)** WGCNA of the correlation of host transcriptome with the rumen wall morphological and development indexes. **(C)** Top KEGG pathway of genes significantly in the MEgreen module. **(D)** Top KEGG pathway of genes significantly in the MEpurple module. The significance of identified KEGG pathways was determined by *p* < 0.05. KEGG, Kyoto Encyclopedia of Genes and Genomes; WGCNA, weighted gene co-expression network analysis. C = basal diet; N = *Saccharomyces cerevisiae* yeast cultures; M = *Kluyveromyces marxianus* yeast cultures; NM = *Saccharomyces cerevisiae* and *Kluyveromyces marxianus* co-cultures yeast cultures.

To explore the relationships between rumen epithelial indices and the effects of NM supplementation on gene expression, WGCNA screening was next performed, leading to the identification of 12 modules of co-expressed genes named after colors (Figures 5A,B). Of these, the MEgreen and MEpurple modules were the most strongly correlated with rumen epithelial measures (papilla length, papilla width, lamina propria thickness, and epithelial thickness). Both the MEgreen (451 genes, 2.6% of total genes) and MEpurple (144 genes, 0.8% of total genes) modules exhibited positive correlations with the NM group whereas they were negatively correlated with the C and M groups.

Additionally, the induction of certain GO terms associated with these modules was evaluated (Table 5). The primary annotated functions attributed to the MEgreen module included translation, mitochondrial electron transport, NADH to ubiquinone, cytoplasmic translation, and glutathione metabolic processes, while the corresponding enriched KEGG pathways included the fatty acid metabolism, oxidative phosphorylation, and FoxO signaling pathways (Figure 5C). DEGs associated with these pathways included *NDUFB9, NDUFB8, NDUFA1, MAPK1, MAPK3, CCNB1, CCND2,* and *ATP5F1D.* The functions of the MEpurple module were primarily associated with protein refolding, cellular response to unfolded protein, chaperone-mediated protein folding requiring cofactor and positive regulation of IκB/NF-κB signaling (Table 5), while corresponding enriched KEGG pathways included the oxidative phosphorylation and fatty acid degradation pathways (Figure 5D). DEGs related to these pathways included *NDUFS3, ACADS,* and *ACAA1.*

**Table 5:** GO enrichment analyses of key hub genes in the MEgreen and MEpurple transcriptomic modules.

Terms	ID	Count	*p*-value
MEgreen			
Translation	GO:0006412	28	0.005
Mitochondrial electron transport, nadh to ubiquinone	GO:0006120	4	0.0067
Cytoplasmic translation	GO:0002181	7	0.0078
Glutathione metabolic process	GO:0006749	5	0.0091
Positive regulation of telomere maintenance via telomerase	GO:0032212	3	0.0127
Cellular response to oxidative stress	GO:0034599	4	0.0155
Regulation of translational initiation	GO:0006446	3	0.0223
Protein folding	GO:0006457	7	0.0251
Mitotic metaphase plate congression	GO:0007080	3	0.0261
Cellular response to lipopolysaccharide	GO:0071222	5	0.0271
Cell cycle	GO:0007049	7	0.0324
Apoptotic process	GO:0006915	8	0.0369
Cholesterol homeostasis	GO:0042632	4	0.0383
Formation of cytoplasmic translation initiation complex	GO:0001732	3	0.0386
One-carbon metabolic process	GO:0006730	3	0.0386
Regulation of Golgi inheritance	GO:0090170	2	0.0389
Caveolin-mediated endocytosis	GO:0072584	2	0.0389
Er to Golgi vesicle-mediated transport	GO:0006888	6	0.0425
Keratinocyte differentiation	GO:0030216	3	0.0432
Thymus development	GO:0048538	3	0.0479
MEpurple			
Protein refolding	GO:0042026	4	0.0011
Cellular response to unfolded protein	GO:0034620	3	0.0052
Chaperone-mediated protein folding requiring cofactor	GO:0051085	3	0.0129
Positive regulation of IκB kinase/NF-κB signaling	GO:0043123	3	0.0377
Neutrophil aggregation	GO:0070488	2	0.0128
Leukocyte migration involved in inflammatory response	GO:0002523	2	0.0191
Fatty acid beta-oxidation using acyl-CoA dehydrogenase	GO:0033539	2	0.0254
Deoxyribonucleotide catabolic process	GO:0009264	2	0.0317

### 3.6 qPCR-based result verification

**Figure 6:**
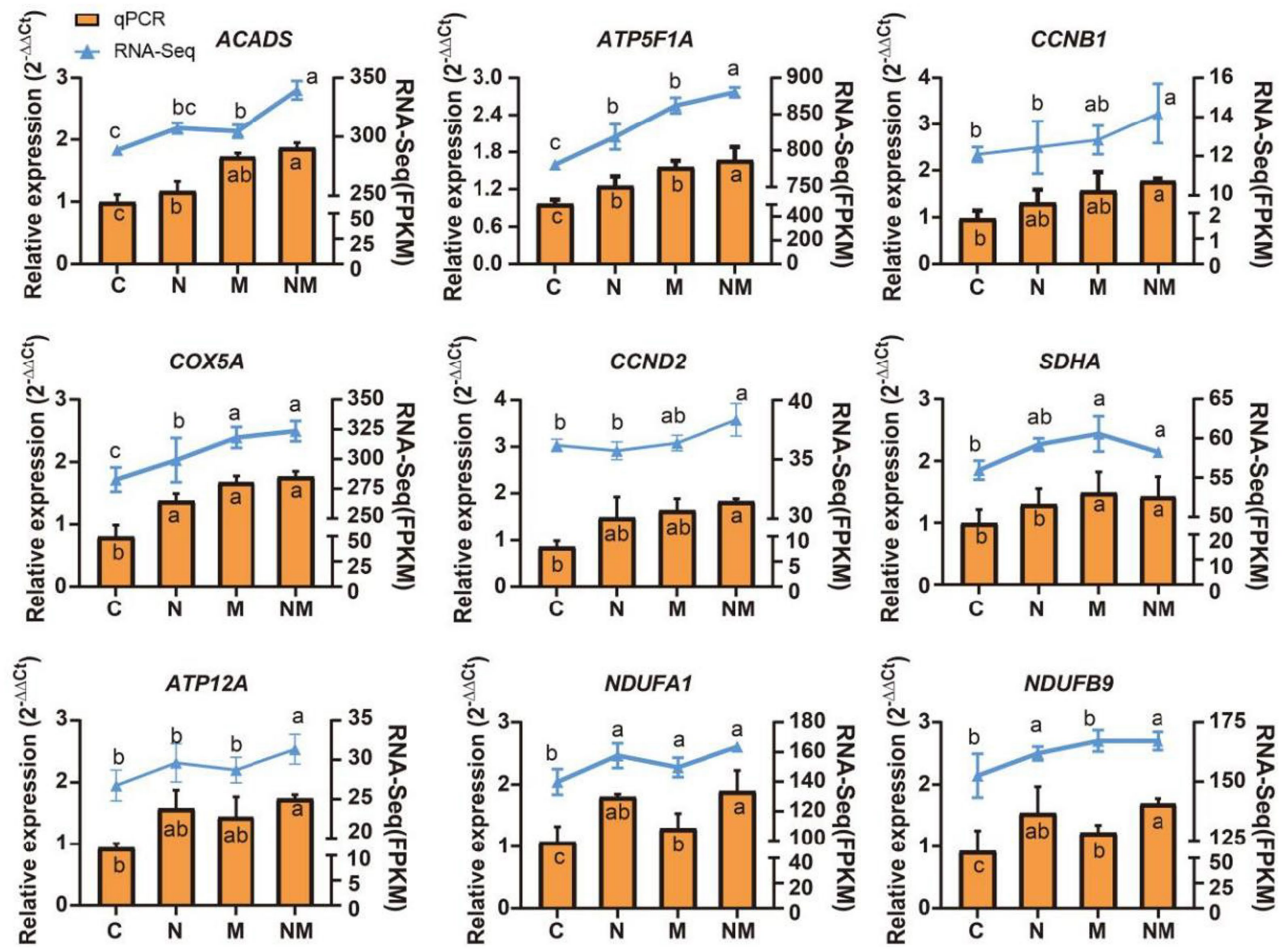
qPCR-based analyses of *ACADS*, *ATP5F1A*, *CCNB1*, *COX5A*, *MAPK1*, *MAPK3*, *MYD88*, *NDUFA1*, and *NDUFB9* expression were used to validate RNA-seq results. a-cindicates the difference between different superindexes within the same index column (*p* < 0.05). C = basal diet; N = *Saccharomyces cerevisiae* yeast cultures; M = *Kluyveromyces marxianus* yeast cultures; NM = *Saccharomyces cerevisiae* and *Kluyveromyces marxianus* co-cultures yeast cultures.

Specific DEGs associated with rumen morphology and the oxidative phosphorylation pathway identified in the WGCNA analyses above were selected for qPCR-based validation, including *NDUFA1, NDUFB9, ATP5F1A, COX5A, CCND2, SDHA, CCNB1, ACADS,* and *ATP12*A, the results showed that the expression pattern was consistent with that of RNA-seq (Figure 6). These analyses revealed that relative to the C group, rumen epithelial tissues from lambs in the N group exhibited increased ACADS, ATP5F1A, COX5A, *NDUFA1* and *NDUFB9* expression (*p* < 0.05), while group M exhibited *ACADS*, *ATP5F1A*, *COX5A*, *SDHA*, *NDUFA1* and *NDUFB9* upregulation (*p* < 0.05), and group NM exhibited the upregulation of *ATP5F1A, CCNB1*, *COX5A*, *NDUFA1*, *CCND2*, *SDHA*, *ATP12A*, *NDUFB9* and *ACADS* (*p* < 0.05).

## 4 Discussion

The development of the rumen is a vital physiologic process in ruminants, as it is responsible for transforming substances that subsequently pass into the intestines, liver, and periphery (38). The application of yeast cultures as a means of enhancing ruminal development has been a recent focus of growing interest (39). Previous studies have shown that dietary supplementation of *S. cerevisiae* and *K. marxianus* yeast co-culture can increase the average daily gain in lambs, and alter the composition and metabolite profiles of the ruminal microbiome (30), but no prior studies have performed transcriptomic analyses exploring the effects of *S. cerevisiae* and *K. marxianus* yeast co-cultures yeast cultures on the morphology and functionality of the rumen wall. The papillae of the rumen contribute to an increase in the surface area with which the rumen wall can interact with the chyme, leading to better nutrient absorption (40). Rumen development is most commonly evaluated based on the width and length of these papillae (41). In the present analyses, the yeast co-cultures contributed to significant beneficial effects on epithelial thickness and papillary width/length in the rumen of lambs in the NM group. Prior reports have noted that the introduction of yeast cultures can lead to improved epithelial thickness (42). This may be attributable to large quantities of nutritionally active substances in the yeast cell walls of NM yeast cultures such as *β*-glucan, mannan, and organic acids which can provide the fuel for the development of the rumen (43).

A series of transcriptomic analyses were conducted to assess changes in the patterns of gene expression in weaned lambs fed an NM-supplemented diet. Through GO enrichment analyses, the DEGs identified in group N were found to be related to the negative regulation of endopeptidase activity, keratinization, and complement activation, classical pathway, in partial agreement with prior results (44, 45). The DEGs in the M and NM groups were associated with defense responses to virus and negative regulation of viral genome replication. These underlying molecular associations and mechanisms require further investigation in the future. KEGG analyses additionally revealed that DEGs associated with the oxidative phosphorylation pathway were significantly enriched for all three analyzed comparisons (C vs. N, C vs. M, C vs. NM, and N vs. NM,). The oxidative phosphorylation process is catalyzed by complexes I, II, III, IV, and V, oxidizing nutrients and producing chemical energy (46), which is used to produce ATP through the phosphorylation of ADP by ATP synthase (47). Mitochondrial oxidative phosphorylation is a hallmark of most aerobic eukaryotes, providing the majority of the energy required by cells (48). Complex I, also referred to as NADH dehydrogenase, is responsible for the conversion of NADH to NAD+. Complex I is the largest enzymatic complex within the electron transport chain (49, 50), consisting of 46 subunits in mammals (51), with these subunits being encoded by both nuclear and mitochondrial genes. Complex I allows for the binding of NADH to the distal portion of its hydrophilic arm, with two electrons being transferred to coenzyme Q after it has bound (52). The reduction of coenzyme Q results in a change in membrane arm conformation, with four proteins being transferred through the membrane (53). Complex I is required for normal cellular function such that, when impaired, defects will arise including conditions such as growth retardation and metabolic disorders (54). Here, the dietary supplementation in group N was associated with *NDUFA1, NDUFS4,* and *NDUFB9* upregulation, while group M exhibited *NDUFA1, NDUFV3, NDUFA10,* and *NDUFA6,* and group NM exhibited the upregulation of *NDUFA1, NDUFA12 NDUFA10, NDUFV1, NDUFV2,* and *NDUFB9*, demonstrating the close link between dietary yeast intake, shifts in the expression of genes related to the first step in the electron transport chain, and the growth and development of the rumen epithelium.

**Figure 7:**
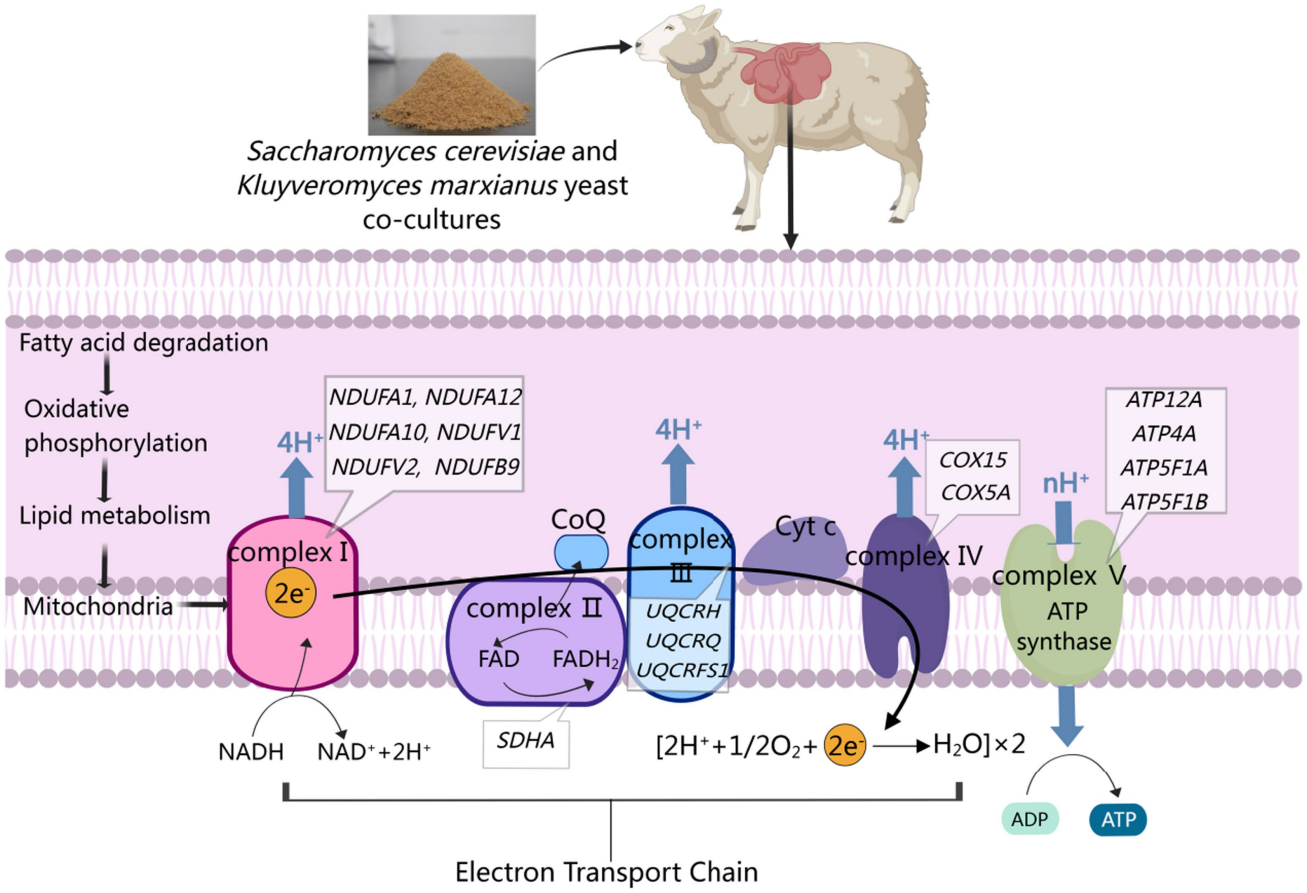
Adding co-cultured yeast to weaned lamb diets can enhance oxidative phosphorylation through the activation of fatty acid degradation, leading to accelerated lipid metabolism, the production of the ATP necessary for rumen growth, and the maintenance of NADH/NAD+ homeostasis within cells.

Complex II, also referred to as succinate dehydrogenase, is composed of the SDHA, SDHB, SDHC, and SDHD subunits (55). It is the only enzymatic complex involved in both the oxidative phosphorylation-related electron transport chain and the mitochondrial TCA cycle through the oxidation of succinate to fumarate (56). In the present study, *SDHA* upregulation was observed in the rumen epithelium from the M and NM groups, indicating that these dietary supplementation practices impacted complex II. Complex III (ubiquinol-cytochrome c reductase) is an essential component of the mitochondrial respiratory chain responsible for transferring electrons to cytochrome C from coenzyme Q (57). This complex is composed of the mitochondrially-encoded MTCYTB and 10 other subunits that are encoded by genes in the nucleus (58). In the current study, upregulation of *UQCRH*, *UQCRQ*, and *UQCRFS1* in the ruminal epithelium upon NM group that the assembly of complex III was affected, and its structure and function were possibly changed. Complex IV (cytochrome C oxidase) received electrons from cytochrome c molecules and transfers electrons to dioxygen molecules to produce H_2_O (59), while also driving the flow of protons across the membrane, leading to an increase in transmembrane electrochemical potential (60). Here, *COX15* and *COX5A* upregulation was noted in the N, M, and NM groups, suggesting that these diets modulated complex IV structural and functional characteristics. Complex V (ATP synthase) can take advantage of this proton gradient to produce ATP using substrates consisting of ADP and Pi (61). Here, the upregulation of *ATP5F1A, ATP5PB,* and *ATP5F1C* was noted in group N, while *ATP5F1A* and *ATPF1B* were upregulated in group M, and group NM exhibited *ATP12A, ATP4A, ATP5F1A,* and *ATP5F1B* upregulation, suggesting that yeast co-cultures can alter this pathway in the context of rumen development by controlling ATP production. Given its efficiency as a means of producing ATP, oxidative phosphorylation plays an essential role in various biological processes (62). The dietary supplementation in group N impacted complexes I, IV, and V, while that in group M affected complexes I, II, IV, and V, and that in group NM impacted complexes I, II, III, IV, and V. These analyses highlighted the ability of co-culture yeast fermentation to promote oxidative phosphorylation within the rumen epithelium in lambs, leading to enhanced ATP production and a more robust intracellular supply of energy that can impact rumen epithelial growth and homeostasis (Figure 7).

In WGCNA analyses probing the link between NM co-culture supplementation and shifts in the morphology of the rumen wall, the MEgreen and MEpurple modules were identified as being most important. GO terms associated with the genes in the MEgreen module related to the development of the rumen epithelium included translation, mitochondrial electron transport, NADH to ubiquinone, and cytoplasmic translation are highlighted as features of rumen epithelial development. Shared DEG analyses also revealed significant changes in the fatty acid metabolism, oxidative phosphorylation, and FoxO signaling pathways associated with the development of the rumen wall. Previously, mitosis has been shown to play an essential role in rumen epithelial growth and development, particularly in phases G1 and G2 (63). Strikingly, the *CCB1* and *CCND2* genes, which are closely linked to the cell cycle, were upregulated in the rumen epithelium of lambs from the NM group. The stability of CCNB1 and CCND2 is dependent on the capacity for ATP biogenesis (64). CCNB1 and CCND2 stabilization during the G1-S transition and G2 phase may thus be linked to NM supplementation. Genes in the MEpurple module were primarily associated with the oxidative phosphorylation and fatty acid degradation pathways. Based on these findings, the yeast cultures in the NM group were speculated to activate fatty acid degradation pathways, promoting oxidative phosphorylation and greater lipid metabolism so as to produce higher levels of ATP necessary to fuel the growth of the rumen epithelium.

## 5 Conclusion

In conclusion, supplementing the diets of weaned lambs with *S. cerevisiae* and *K. marxianus* yeast co-cultures can drive improvements in rumen epithelial development and morphology. These transcriptomic analyses demonstrated that the utilized yeast co-cultures were able to promote enhanced oxidative phosphorylation through the activation of the fatty acid degradation pathway, resulting in enhanced lipid metabolism such that higher levels of ATP necessary for rumen development were generated while sustaining appropriate NADH/NAD+ homeostasis within cells. These results emphasize the benefits that yeast co-cultures can have on the development of the rumen, offering new insight into the gene expression change that control this developmental process.

## Data Availability

The data presented in this study are openly available in Sequence Read Archive at https://www.ncbi.nlm.nih.gov/sra (accessed on 10 December 2023), reference number PRJNA1031991.

## Footnotes

^1^ https://www.genescloud.cn
