# Supplementary material for: Transcriptomic characterization of the functional and morphological development of the rumen wall in weaned lambs fed a diet containing yeast co-cultures of Saccharomyces cerevisiae and Kluyveromyces marxianus
Source: Front Vet Sci. 2025 Jan 22;12:1510689. doi: 10.3389/fvets.2025.1510689 (PMC11794207; doi:10.3389/fvets.2025.1510689)
Supplement: Supplementary file 1 [file Table_1.DOCX]

**Table S1** Nutrient composition of mixed feed and yeast culture (feed-based).

| Item | MF | N | M | NM |
| --- | --- | --- | --- | --- |
| Crude protein, % | 19.23 | 20.35 | 20.55 | 20.39 |
| Dry matter, % | 92.04 | 93.23 | 92.58 | 93.21 |
| Neutral detergent fiber, % | 34.11 | 32.45 | 33.72 | 34.23 |
| Acid detergent fiber, % | 20.02 | 19.42 | 19.23 | 19.97 |
| Live yeast cells, CFU/g |  | 6.8×10^4^ | 1.5×10^4^ | 7.2×10^4^ |
| Lactic acid, mmol/kg |  | 371.13 | 391.57 | 380.31 |

MF=mixed feed; N=*Saccharomyces cerevisiae* yeast cultures; M=*Kluyveromyces marxianus* yeast cultures; NM=*Saccharomyces cerevisiae* and *Kluyveromyces marxianus* co-cultures yeast cultures.

**Figure S1**


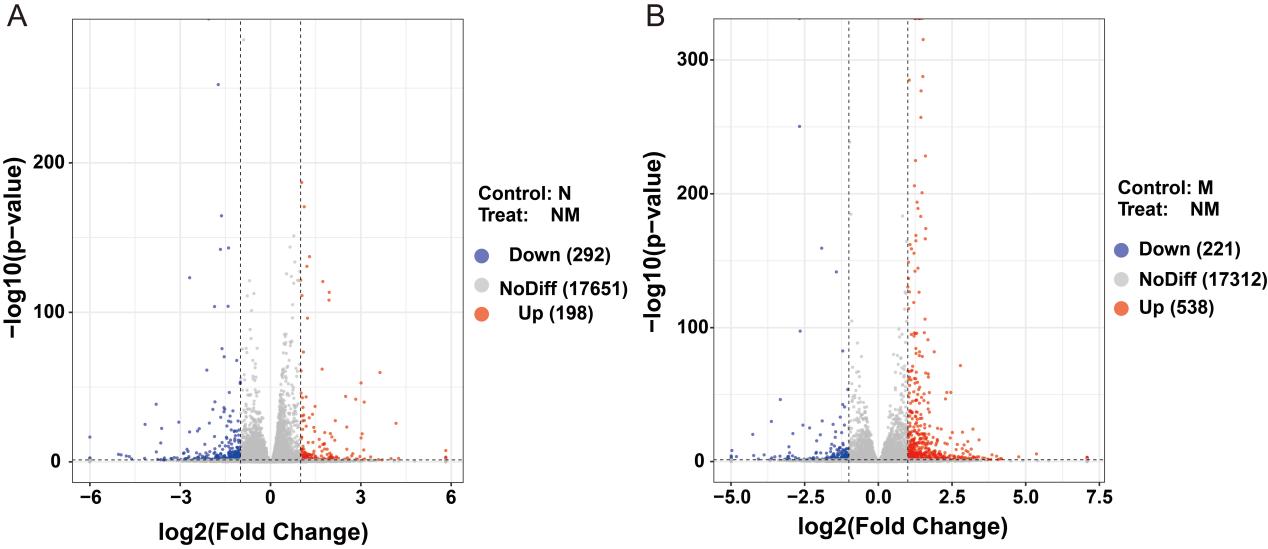


**Figure S1.** Differentially expressed gene identification in the ruminal epithelium for the three different group comparisons. (A) N vs. NM, (B) M vs. NM. In the generated volcano plots, the X- and Y-axis respectively represent log2 fold change and log10 *P*-values, with blue, red, and gray dots indicating downregulated DEGs, upregulated DEGs, and non-DEGs, respectively. N=*Saccharomyces cerevisiae* yeast cultures; M=*Kluyveromyces marxianus* yeast cultures; NM=*Saccharomyces cerevisiae* and *Kluyveromyces marxianus* co-cultures yeast cultures

**FigureS2**
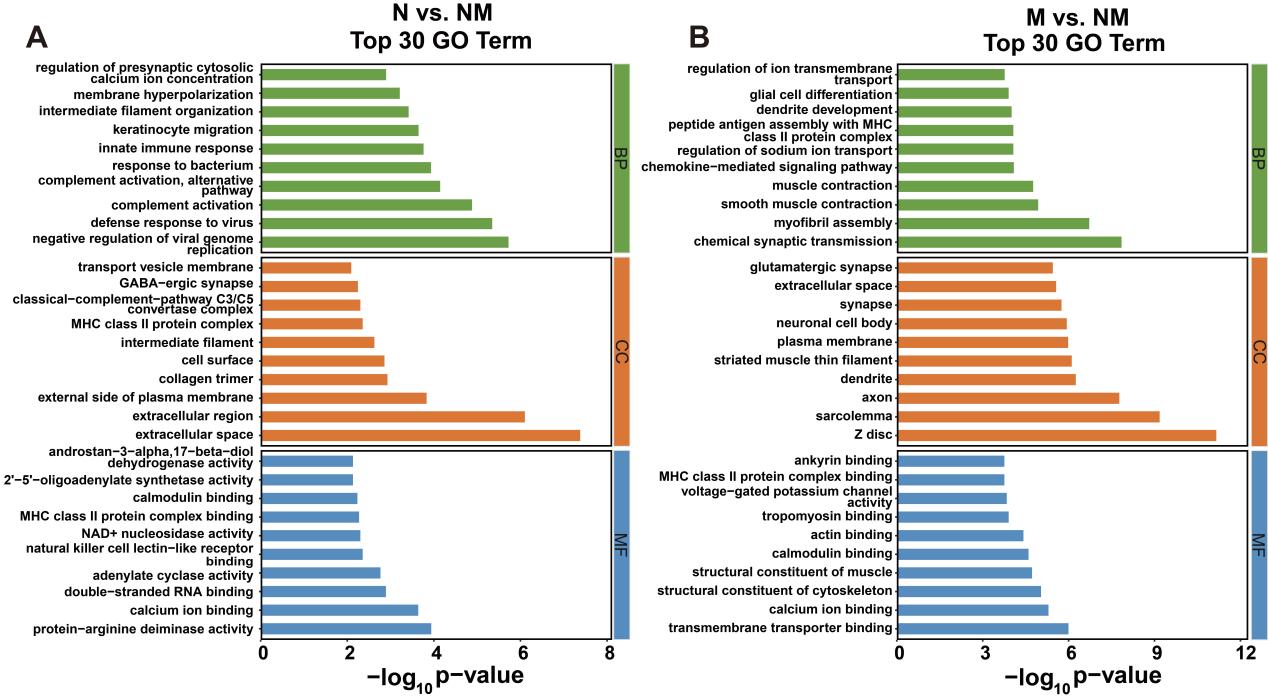


**Figure S2.** Analyses of DEG enrichment in specific GO terms. The top 30 biological process, cellular component, and molecular function terms are shown (*P* < 0.05; the DEGs number of GO terms was >2).

N=*Saccharomyces cerevisiae* yeast cultures; M=*Kluyveromyces marxianus* yeast cultures; NM=*Saccharomyces cerevisiae* and *Kluyveromyces marxianus* co-cultures yeast cultures.

**FigureS3**


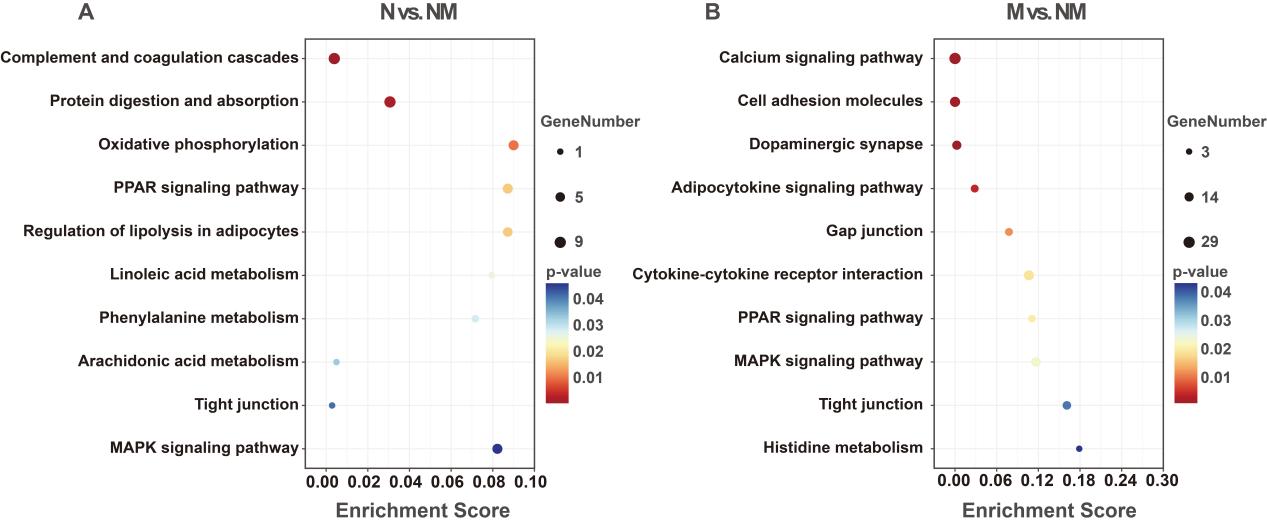


**Figure S3.** Analysis of DEG enrichment in KEGG pathways. Pathways and enrichment scores are respectively shown on the Y- and X-axes. Bubble sizes and colors correspond to significance levels and number of genes in each pathway, respectively (*P* < 0.05). (A) N vs. NM. (B) M vs. NM.

N=*Saccharomyces cerevisiae* yeast cultures; M=*Kluyveromyces marxianus* yeast cultures; NM=*Saccharomyces cerevisiae* and *Kluyveromyces marxianus* co-cultures yeast cultures.
